# Pretransplantation seroreactivity in kidney donors and recipients as a predictive factor for posttransplant BKPyV-DNAemia

**DOI:** 10.3389/fimmu.2022.929946

**Published:** 2022-07-25

**Authors:** Martina Saláková, Viera Ludvíková, Eva Hamšíková, Marie Kolářová, Vojtěch Šroller, Ondřej Viklický, Mariana Wohlfahrtová

**Affiliations:** ^1^ Department of Genetics and Microbiology, Faculty of Science, Charles University, Prague, Czechia; ^2^ National Reference Laboratory for Papillomaviruses and Polyomaviruses, Institute of Hematology and Blood Transfusion, Prague, Czechia; ^3^ Department of Nephrology, Transplant Centre, Institute for Clinical and Experimental Medicine, Prague, Czechia

**Keywords:** BK polyomavirus (BKPyV), BKPyV-associated nephropathy, kidney transplantation, seroreactivity, seroprevalence

## Abstract

BK polyomavirus (BKPyV) often reactivates after kidney transplantation, causing BKPyV-associated nephropathy (BKPyVAN) in 1%–10% of cases with a potential detrimental effect on allograft survival. Kidney transplant recipients are regularly screened for BKPyV DNA in plasma. As this strategy may not always reduce the risk of BKPyVAN, other predictive markers are needed. To evaluate the role of pretransplant BKPyV-specific antibody, 210 kidney transplant recipients and 130 donors were screened for BKPyV DNA and BKPyV-specific antibodies. We found that the donor BKPyV immunoglobulin G (IgG) seroprevalence and antibody level were strongly associated with BKPyV-DNAemia and BKPyVAN, although multivariant analysis found the presence of anti-BKPyV-specific antibodies as a predictive factor only for BKPyV-DNAemia. The pretransplant recipient status had no effect on posttransplant BKPyV-DNAemia and BKVAN. BKPyV IgG levels remained stable in BKPyV-negative recipients during 1-year follow-up, while a considerable increase was observed in BKPyV-positive patients. The presence of anti-BKPyV-specific antibodies in kidney allograft donors is a good and reliable predictive marker for posttransplant BKPyV replication with relevance to risk stratification in transplant recipients.

## Introduction

BK polyomavirus (BKPyV) is a small non-enveloped DNA virus belonging to the Polyomaviridae family. The virus is widespread, with a seroprevalence of more than 70% among the general population (1). Infection usually occurs in early childhood, and the virus establishes lifelong persistence. After kidney transplantation (KT), BKPyV often reactivates, leading to a high viral load in the urine in 30%–60% of the recipients, followed by the presence of BKPyV in plasma (BKPyV-DNAemia) in almost half of the recipients 2–6 weeks later. Reactivation results in BKPyV-associated nephropathy (BKPyVAN) in up to 10% of patients at risk of renal allograft dysfunction and in graft loss in 15%– 50% of cases (2). The definitive diagnosis of BKPyVAN is based on the morphological evidence of polyomavirus replication in the kidney tissue by biopsy. Morphologically apparent polyomavirus injury of renal allograft tissue is confirmed by immunohistochemistry using cross‐reacting monoclonal LTag antibodies of the Simian polyomavirus (SV40). There is no specific antiviral treatment; immunosuppression is standardly reduced or discontinued in patients with a high BKPyV load. Current guidelines recommend regular screening to detect BKPyV-DNAemia after renal transplantation. Sustained detection of BKPyV in the plasma or viral load in the plasma of >10^4^ genome copies per milliliter is associated with a BKPyVAN risk (1).

The adaptive immune response plays a key role in clearing BKPyV infection. The role of T cells is the most important, and among them, the CD8+ T cells predominate over CD4+ T cells (3, 4). The impact of BKPyV-specific antibodies seem to be less clear. BKPyV seropositivity is unlikely to completely protect the recipient from viral replication and BKPyVAN, but the absence or low level of specific antibodies to BKPyV may pose a higher risk of BKPyV-DNAemia and nephropathy (5, 6). On the other hand, high titers of donors’ BKPyV-specific antibodies was associated with a higher risk of recipients’ BKPyV-DNAemia and BKPyVAN development (6).

The strategies to reduce immunosuppression may not always eliminate the risk of BKPyVAN, and some recipients do not exceed the viral load threshold and are misclassified (7). Therefore, it is important to refine the identification of patients at higher risk of developing nephropathy. Early identification of patients at risk of BKPyV reactivation is also essential. In this study, we evaluated the risk factors that influence BKPyV reactivation and the development of BKPyVAN. We focused on the role of BKPyV-specific antibody titers in kidney donors and recipients along with other clinical characteristics to assess serology for promising pretransplant markers.

## Materials and methods

### Study population

Two hundred and thirty patients who underwent KT at the Institute for Clinical and Experimental Medicine, Prague between July 2017 and July 2018 were enrolled in this study. Twenty kidney recipients were excluded from the follow-up due to early graftectomy, exitus letalis, and failed transplantation or because of the termination of commuting to the Institute for Clinical and Experimental Medicine. Peripheral blood samples were collected from kidney recipients at the day of transplantation (at baseline, median 0 day, 95% CI: 0) and at posttransplant month 1 (M1, median 26, 95% CI: 26–27), 3 (M3, median 86, 95% CI: 85–87), 6 (M6, median 178, 95% CI: 176–180), and 12 (M12, median 360, 95% CI: 357–363) after transplantation. One hundred and thirty peripheral blood samples from kidney donors were included in the study. Plasma isolated from peripheral blood was aliquoted and stored at -80°C. The study received official institutional and ethical approval (number G-16-06-27), and all patients signed the written informed consent form. Renal function was determined by serum creatinine and estimated glomerular filtration rate (eGFR) level measurement and by monitoring proteinuria. Patients’ demographic and clinical characteristics were collected from medical records. Data are shown in **Table 1**.

**Table 1:** Patients’ demographic and clinical characteristics.

Characteristics	Study cohort	BKPyV negative	BKPyV-DNAemia viremia	
	n=210	n=157	n=53	p
Recipient gender—female (n, %)	76 (36.2)	55 (35.0)	21 (39.6)	0.621
Recipient age (mean, SD)	54 (13.3)	53 (13)	54 (13)	0.613
Type of dialysis (n, %)				
None—preemptive Tx	17 (8.1)	12 (7.6)	5 (9.4)	0.915
Hemodialysis	152 (72.4)	114 (72.6)	38 (71.7)	
PD	41 (19.5)	31 (19.7)	10 (18.9)	
Dialysis vintage (mean, SD)	2.5 (2.1)	2.4 (2.2)	2.5(2.0)	0.718
Retransplantation (n, %)	28 (13.3)	20 (12.7)	8 (15.1)	0.646
Rejection in previous Tx (n, %)	19 (67.9)	13 (65.0)	6 (75.0)	1.0
Peak PRA (mean, SD)	21(24)	21.4(25)	19.5(22)	0.937
HLA mismatch (mean, SD)	3 (1.4)	3.3 (1.4)	3.4 (1.4)	0.648
Positive DSA (n, %)	18 (8.6)	14 (8.9)	6 (11.3)	0.595
Donor age (years, SD)	53 (15)	54 (14)	52 (18)	0.766
Donor gender - female (n, %)	89 (42.4)	66 (42.0)	23 (43.4)	0.874
Living donor (n, %)	39 (18.6)	29 (18.5)	10 (18.9)	1.0
ECD (n, %)	116 (55.2)	89 (56.7)	27 (50.9)	0.524
CMV prophylaxis (n, %)	167 (79.5)	130 (82.8)	37 (69.8)	0.050
CMV mismatch (n, %)	32 (15.2)	21 (13.4)	11 (20.8)	0.268
DGF (n, %)	59 (28.1)	51 (32.5)	8 (15.1)	0.014*
Induction treatment (n, %)				
Basiliximab	59 (28.1)	39 (24.8)	20 (37.7)	0.038*
rATG	150 (71.4)	118 (75.2)	32 (60.4)	
Alemtuzumab	1 (0.5)	0 (0)	1 (1.9)	
1-year graft survival (n, %)	207 (98.6)	154 (98.1)	52 (98.1)	0.991
Serum creatinine at M12 (mean, SD)	133.2 (91.0)	131.6 (83.7)	138.0 (110.7)	0.787
eGFR at M12 (mean, SD)	0.93 (0.33)	0.93 (0.32)	0.94 (0.37)	0.997
Acute rejections (n, %)	28 (13.3)	21 (13.4)	7 (13.2)	1.0
Chronic rejections (n, %)	8 (3.8)	6 (3.8)	2 (3.8)	1.0

### BK polyomavirus DNA detection

DNA was extracted from 200 μl of plasma with the DNA mini kit (Qiagen, Hilden, Germany) according to the manufacturer’s instructions.

Quantitative polymerase chain reaction (qPCR) analysis was performed using the Biorad CFX96 Real-Time PCR system (Biorad Laboratories, Hercules, CA, USA). Each qPCR reaction contained a 1 × PCR buffer, 5 mM MgCl_2_, 200μM deoxynucleotide triphosphates (dNTPs)Prevalence of BK polyomavirus (BKPyV)-DNAemia in recipients, 0.5μM concentration each of forward and reverse primers and 0.25μM probe, 0.25 U HotStar Taq Polymerase (Qiagen, Hilden, Germany), and the DNA template. The primers and probe used in this study were selected from the manuscript of Hoffman et al. (8) and were evaluated in INSTANDs, e.V. The cycling conditions were: an initial denaturation step at 95°C for 15 min, followed by 45 cycles with denaturation at 95°C for 15 s and annealing and extension at 60°C for 60 s. The analytical sensitivities of qPCRs were 1 copy of plasmid standard per assay. The contamination of the PCR was checked by including a negative sample and a sample with distilled water in each run. Each qPCR reaction was run in triplicate. For the analysis, the Biorad Maestro software was used (Biorad Laboratories, Hercules, CA, USA).

BKPyV-DNAemia was defined as virus detection in plasma. Presumptive BKPyVAN was defined as >10^4^ copies/ml; lower level (<10^4^ copies/ml) was designated as low BKPyV-DNAemia. The highest viral load was considered to be the peak BKPyV DNA plasma viral load measured in 1-year follow-up. Sustained DNAemia was defined as two or more consecutive positive plasma samples, while transient DNAemia refers to the detection of BKPyV DNA in a single sample.

### BK polyomavirus enzyme-linked immunosorbent assay

A serological assay was performed as described previously, using the BKPyV VP1 virus-like particles (VLPs) produced in the Bac-to-Bac Baculovirus Expression System (Invitrogen, Basel, Switzerland) (9). The VP1 gene corresponded to genotype 1b. The BKPyV IgG antibodies were detected by an indirect enzyme-linked immunosorbent assay (ELISA) (10). Briefly, ELISA plates coated with purified BKPyV VP1 VLPs were loaded with serum samples (diluted to 1:100) in duplicate, and the bound antibodies were detected using donkey anti-human IgG conjugated with peroxidase (Jackson ImmunoResearch Laboratories, Inc, West Grove, PA, USA) and visualized by o-phenylenediamine. The optical density (OD) was measured at 492 and 630 nm by an Infinite 200 microplate reader (TECAN, Männedorf, Switzerland). The same control plasma samples known to be positive or negative were tested with each ELISA plate. An optical density cutoff (CO) value was determined as the mean of the negative control plus two standard deviations (2SDs). The ELISA result was the ratio of the absorbance of a sample to the respective CO value (OD index). Samples with the OD index values of ≤1.0 were considered not reactive.

### Statistical analysis

Data were analyzed with Graph Pad, version 6 (GraphPad Software, San Diego, CA, USA) and IBM SPSS Statistics software version 28 (IBM Corp., Armonk, NY, USA). Differences were evaluated using the chi-square test, Fisher exact test, Student’s t-test, Kruskal–Wallis test, or Mann–Whitney U test, as appropriate to compare variables between groups. Univariate and multivariate regression analyses were performed to determine which baseline covariates affected the development of plasma BKPyV-DNAemia and biopsy-proven BKPyVAN. For all tests in this study, a p-value of <0.05 in a two-sided test was considered statistically significant.

## Results

### Prevalence of BK polyomavirus DNA

In total, 210 plasma samples from the recipients’ cohort were available for analysis. No plasma sample was positive for BKPyV DNA prior to transplantation.

After transplantation (time points M1 to M12), 53 (53/210, 25.2%) were positive for BKPyV DNA in plasma samples (BKPyV-DNAemia). Presumptive BKPyVAN was detected in 32 recipients (15.2%). One hundred and fifty-seven recipients (157/210, 74.8%) remained plasma negative throughout the whole follow-up (BKPyV negative).

**Figure 1:**
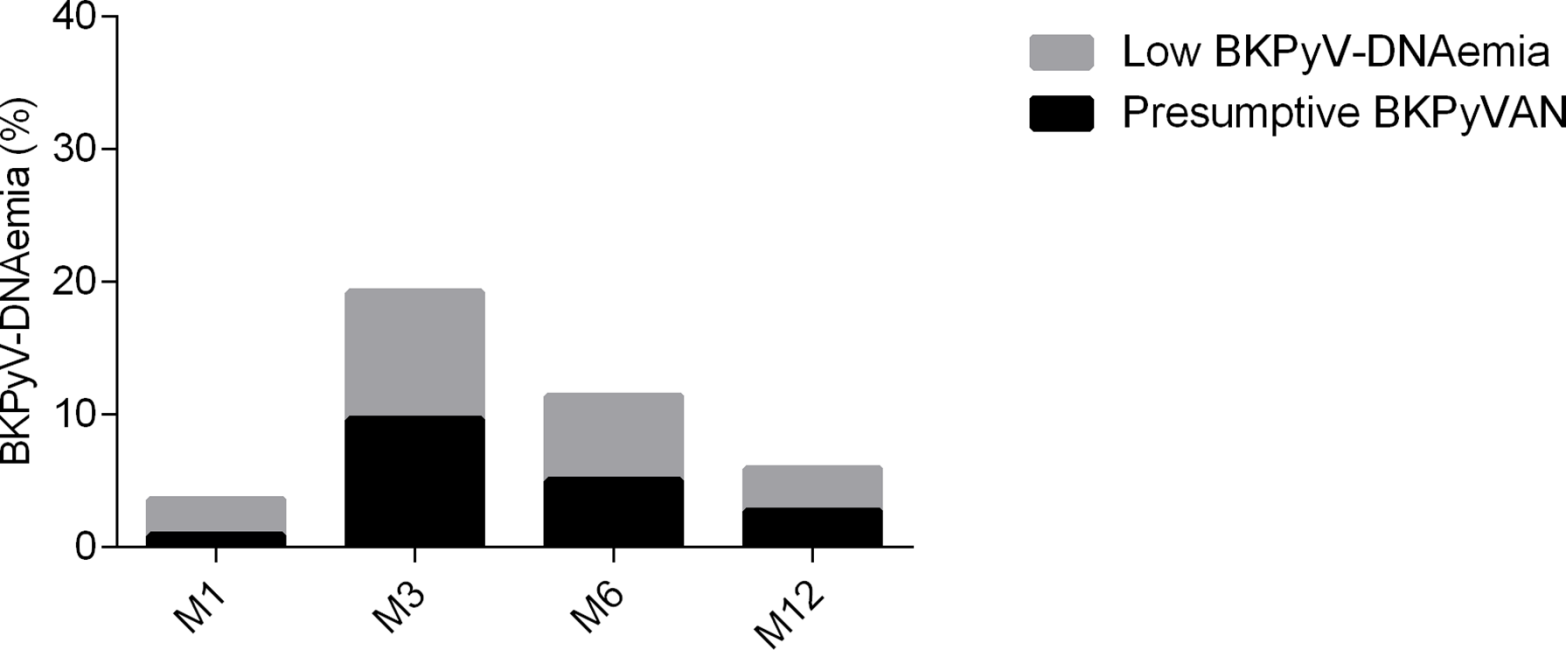
Prevalence of BK polyomavirus (BKPyV)-DNAemia in recipients during the follow-up. Presumptive BKPyVAN was defined as >10^4^ copies/ml, viral load <10^4^ copies/ml viremia as low BKPyV-DNAemia. Legend: BKPyV-DNAemia after transplantation at month 1 (M1), 3 (M3), 6 (M6), and 12 (M12).

Presumptive BKPyVAN was found in 3.6%, 19.1%, 11.4%, and 5.9% of recipients at M1, M3, M6, and M12, respectively (**Figure 1**). Sustained BKPyV-DNAemia was observed in 22 (41.5%) and transient BKPyV-DNAemia in 30 recipients (58.5%).

None of the donors had detectable BKPyV DNA in blood.

### Recipients’ pretransplant and posttransplant serology

**Figure 2:**
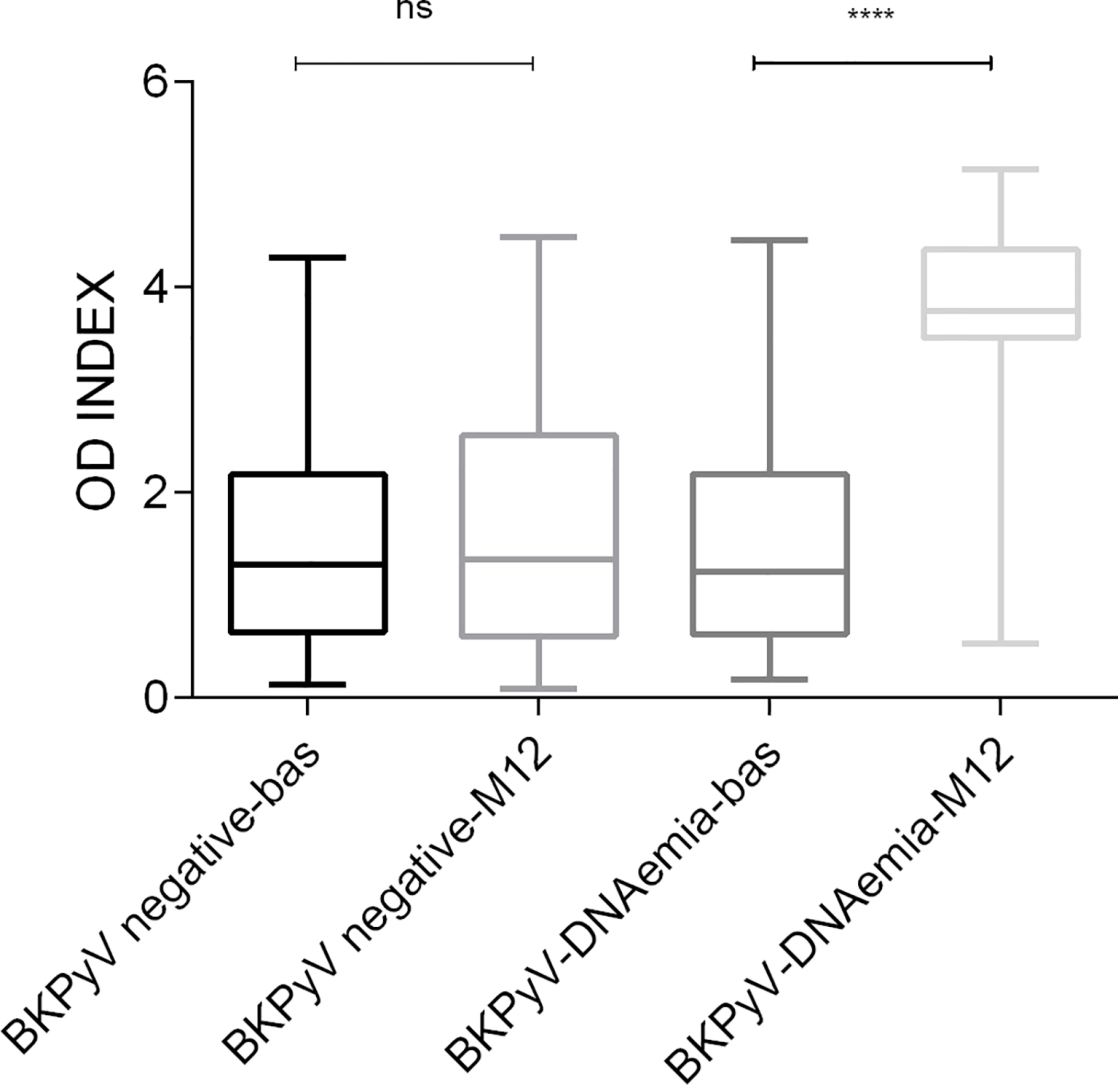
BKPyV IgG seroreactivity among kidney transplant recipients according to BKPyV DNA detection before transplantation (baseline–bas) and at one-year follow-up (M12). Legend: ****p < 0.0001, ns non-significant difference p=0.623, OD INDEX—the ratio of the absorbance of a sample to the respective CO value. bas—baseline, before transplantation; M12—month 12 after transplantation.

The seroprevalence at baseline among all recipients was 61.9%, with the level of antibodies represented by the mean of the OD index of 1.5 ± 1.0. The pretransplant seroprevalence did not differ between recipients who developed posttransplant plasma BKPyV-DNAemia (60.4%) and those who stayed plasma BKPyV negative (62.4%). The baseline BKPyV seroreactivity, in relation to BKPyV replication during the follow-up, was comparable in both groups (p=0.634, **Table 2**; **Figure 2**).

**Table 2:** BKPyV IgG seroreactivity represented by the mean OD index at the time points of 1-year follow-up.

	All recipients	BKPyV negative	BKPyV-DNAemia	p
	(n=210)	(n=157)	(n=53)	
Baseline seroprevalence	130 (61.9%)	98 (62.4%)	32 (60.4%)	0.870
M12 seroprevalence	144 (68.6%)	93 (59.2%)	52 (98.1%)	<0.0001
BKPyV seroreactivity, mean OD index (SD)				
at baseline	1.5 (1.0)	1.5 (1.0)	1.5 (1.0)	0.634
M1	1.4 (0.88)	1.4 (0.88)	1.4 (0.87)	0.755
M3	1.7 (1.1)	1.4 (0.94)	2.0 (1.4)	<0.0001
M6	2.0 (1.4)	1.5 (1.1)	3.5 (1.1)	<0.0001
M12	2.2 (1.5)	1.7 (1.2)	3.7 (1.0)	<0.0001

The seroprevalence at 1 year after transplantation increased to 68.6% in all recipients. The posttransplant OD index increased significantly from the baseline (mean OD index of 1.5 ± 1.0 at the baseline and 2.2 ± 1.5 at M12, p< 0.0001) (**Table 2**). The seropositivity differed significantly between patients according to their BKPyV posttransplant status. In the BKPyV- positive group, the seroprevalence increased to 98.1% (52/53) with a mean OD index of 3.7 ± 1.0, whereas in the BKPyV-negative group, it remained stable (59.2% 93/157) with a mean OD index of 1.7 ± 1.2 (**Table 2**). The only posttransplant seronegative recipient from the BKPyV-DNAemic group was BKPyV DNA plasma positive at the end of the study (M12). The BKPyV seroreactivity did not differ between BKPyV-negative recipients at the time of transplantation and at 1-year follow-up (OD indices of 1.5 ± 1.0 and 1.7 ± 1.2, respectively, p=0.623). In recipients with BKPyV-DNAemia, mean seroreactivity increased more than twofold (OD indices of 1.5 ± 1.0 and 3.7 ± 1.0, respectively, p < 0.0001) (**Figure 2**).

**Figure 3:**
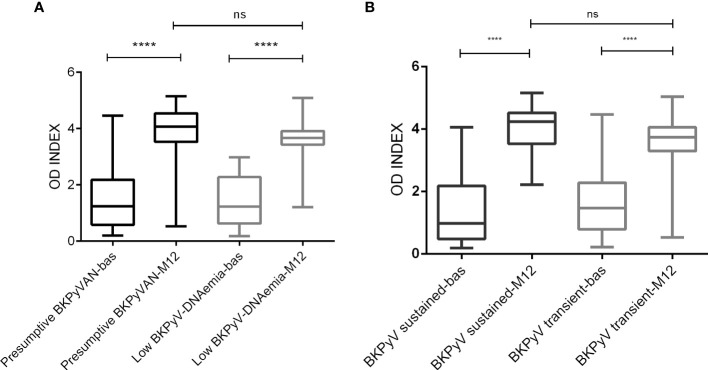
BKPyV IgG seroreactivity among viremic kidney transplant recipients according to the viral load **(A)** and length of BKPyV DNA detection **(B)**. **(A)**. Legend: **** p < 0.0001, ns non-significant difference p=0.0527. OD INDEX - the ratio of the absorbance of a sample to the respective CO value, bas – baseline, before transplantation, M12 – month 12 after transplantation. Presumptive BKPyVAN was defined as >10^4^ copies/ml, viral load <10^4^ copies/ml viremia as low BKPyV-DNAemia, **(B)**. Legend: **** p < 0.0001, ns, non-significant difference; p=0.0777. OD INDEX—the ratio of the absorbance of a sample to the respective CO value, bas—baseline, before transplantation, M12—month 12 after transplantation. Sustained DNAemia was defined as two or more consecutive positive plasma samples, while transient DNAemia refers to the detection of BKPyV DNA in a single sample.

We investigated the effect of the BKPyV viral load in plasma on anti-BKPyV IgG levels at M12. The presumptive BKPyVAN patients had a slightly higher mean OD index compared to the low BKPyV-DNAemic patients (OD indices of 3.8 ± 1.1 and 3.5 ± 0.85, respectively, p=0.0527, **Figure 3A**). The mean OD index of the recipients with sustained BKPyV-DNAemia was also slightly higher compared to that of the recipients with transient BKPyV-DNAemia; this difference was not statistically significant (OD indices of 4.0 ± 0.68 and 3.5 ± 1.2, respectively, p=0.0777, **Figure 3B**).

### The impact of recipients’ pretransplant seroreactivity on posttransplant BK polyomavirus-DNAemia

A recipient’s pretransplant seronegativity was not associated with a significant risk of developing plasma BKPyV-DNAemia. The seronegative recipients (R-) developed viremia in 26.3% (21/80) during the 1-year follow-up while the seropositive recipients (R+) in 24.6% (32/130, p=0.870). The pretransplant levels of antibodies in seropositive recipients did not predict posttransplant BKPyV-DNAemia. The mean OD index was 2.1 ± 0.86 for BKPyV-DNAemic patients and 2.1 ± 0.78 for BKPyV-negative patients (p=0.631).

### The impact of donors’ seroreactivity on posttransplant BK polyomavirus-DNAemia

**Figure 4:**
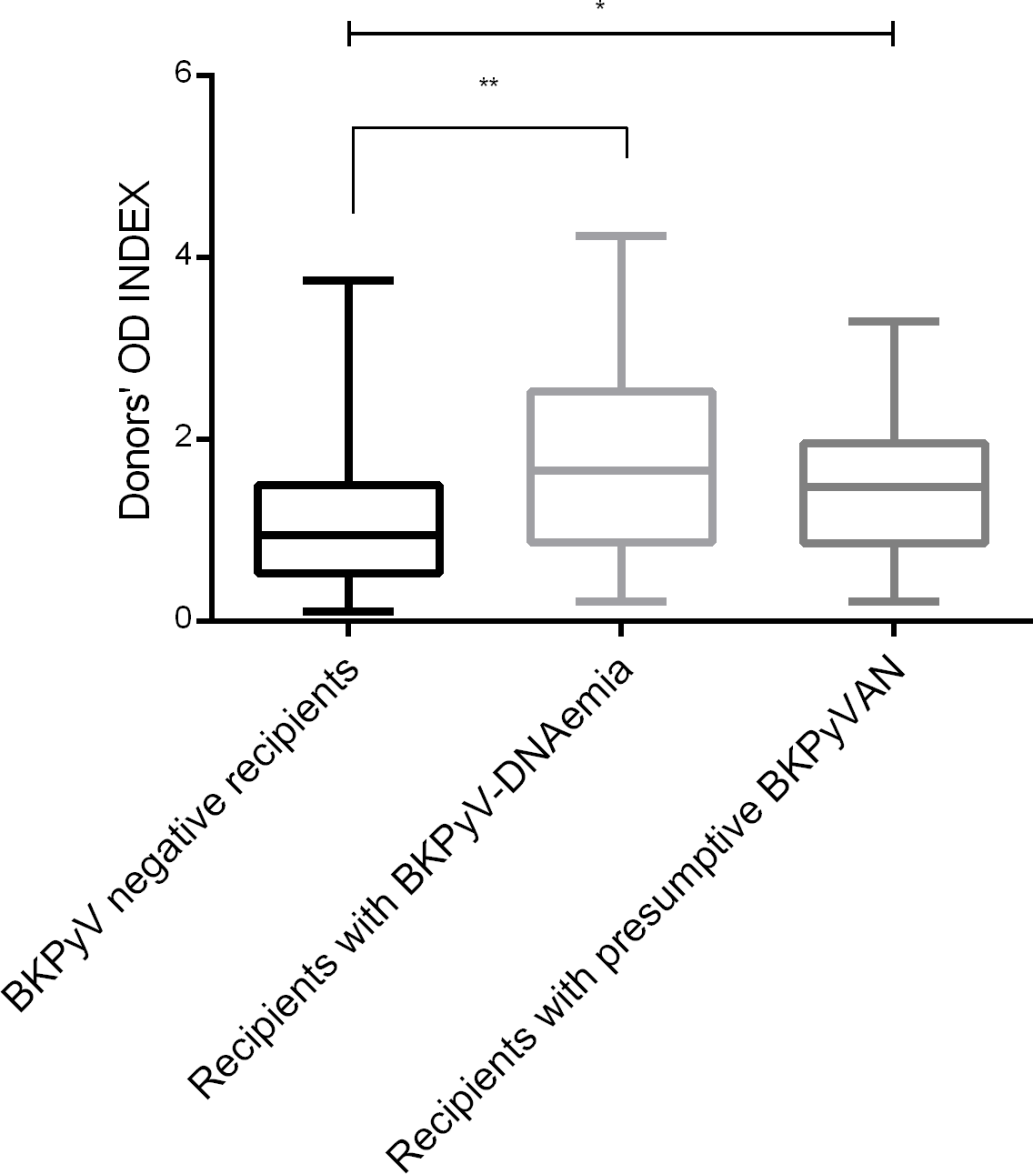
Pretransplant BKPyV seroreactivity in kidney allograft donors stratified by posttransplant BKPyV-DNAemia. Legend: ** p=0.0011, * p=0.026. Donors’ OD INDEX represents the ratio of the absorbance of a sample to the respective CO value; donors were divided according to the posttransplant recipients’ BKPyV status.

One hundred and thirty donor plasma samples were tested for the presence of BKPyV-specific IgG antibodies. The seroprevalence among donors was 53.8%, with the mean OD index of 1.3 ± 0.89. BKPyV-DNAemia was developed more frequently in kidney recipients from seropositive donors (D+) compared to seronegative donors (D-) (31.3% versus 13.7%, p= 0.013). Patients with presumptive BKPyVAN tended to be more common among recipients who received kidney from seropositive donors, but the difference was not statistically significant (20.2% versus 9.5%, p=0.076). **Figure 4** shows the OD index of the donors’ groups stratified by the recipients’ posttransplant status. Statistically significantly higher OD indices were observed in the donors of recipients with posttransplant BKPyV-DNAemia (p=0.0011) and presumptive BKPyVAN (p=0.026).

When considering the pairs of donors and recipients for pretransplant seroprevalence and posttransplant BKPyV-DNAemia, the results showed increasing frequency from 6.7% (2/30) for the D-/R- group and 19.5% (8/41) for the D-/R+ group to 28% (14/50) for the D+/R+ group and 36.4% (12/33) for the D+/R- group (p=0.033).

### Risk factors for biopsy-proven BK polyomavirus-associated nephropathy and BK polyomavirus-DNAemia

**Figure 5:**
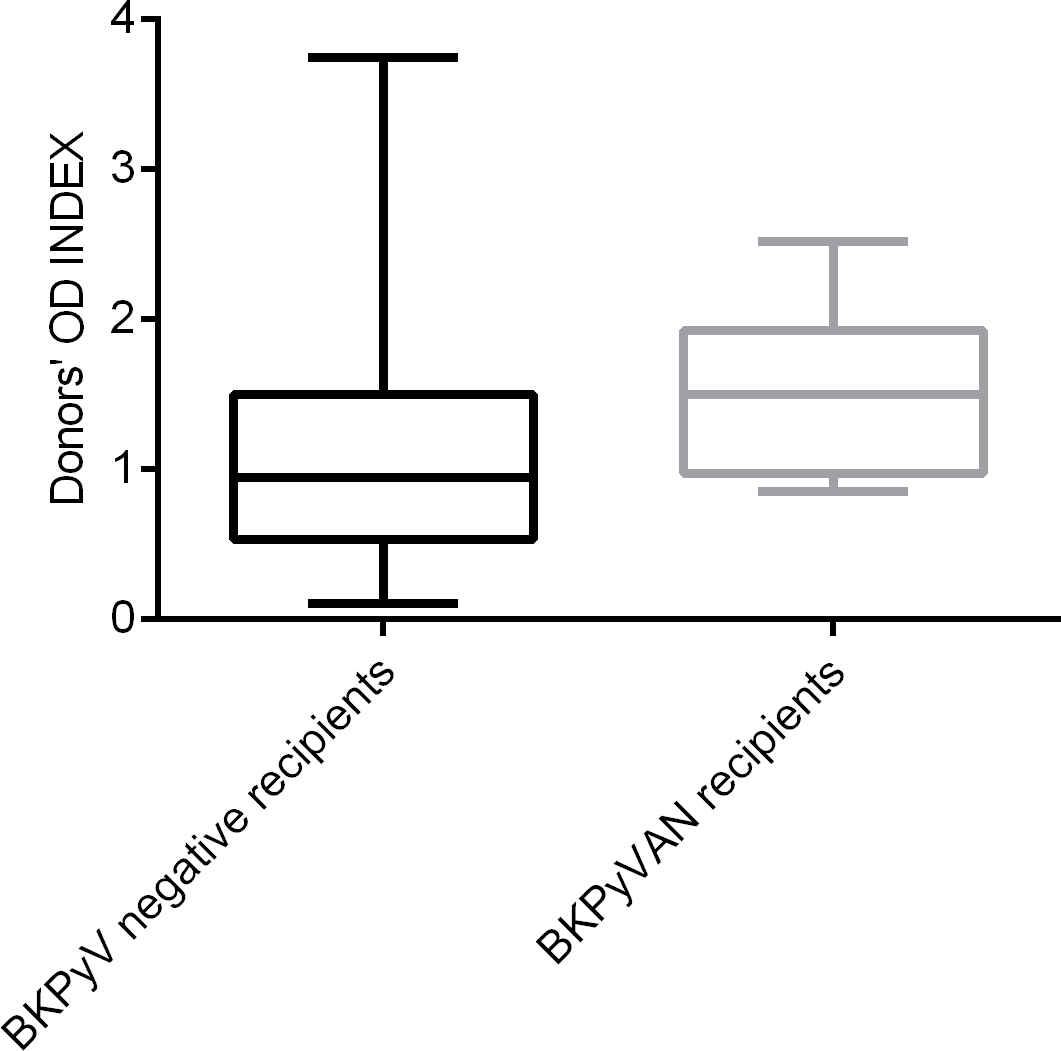
Pretransplant BKPyV seroreactivity among kidney allograft donors of BKPyV- negative recipients and those with posttranplant BKPyVAN. Legend: p=0.058, donors’ OD INDEX represent the ratio of the absorbance of a sample to the respective CO value; donors were divided according to posttransplant BKVAN.

**Figure 6:**
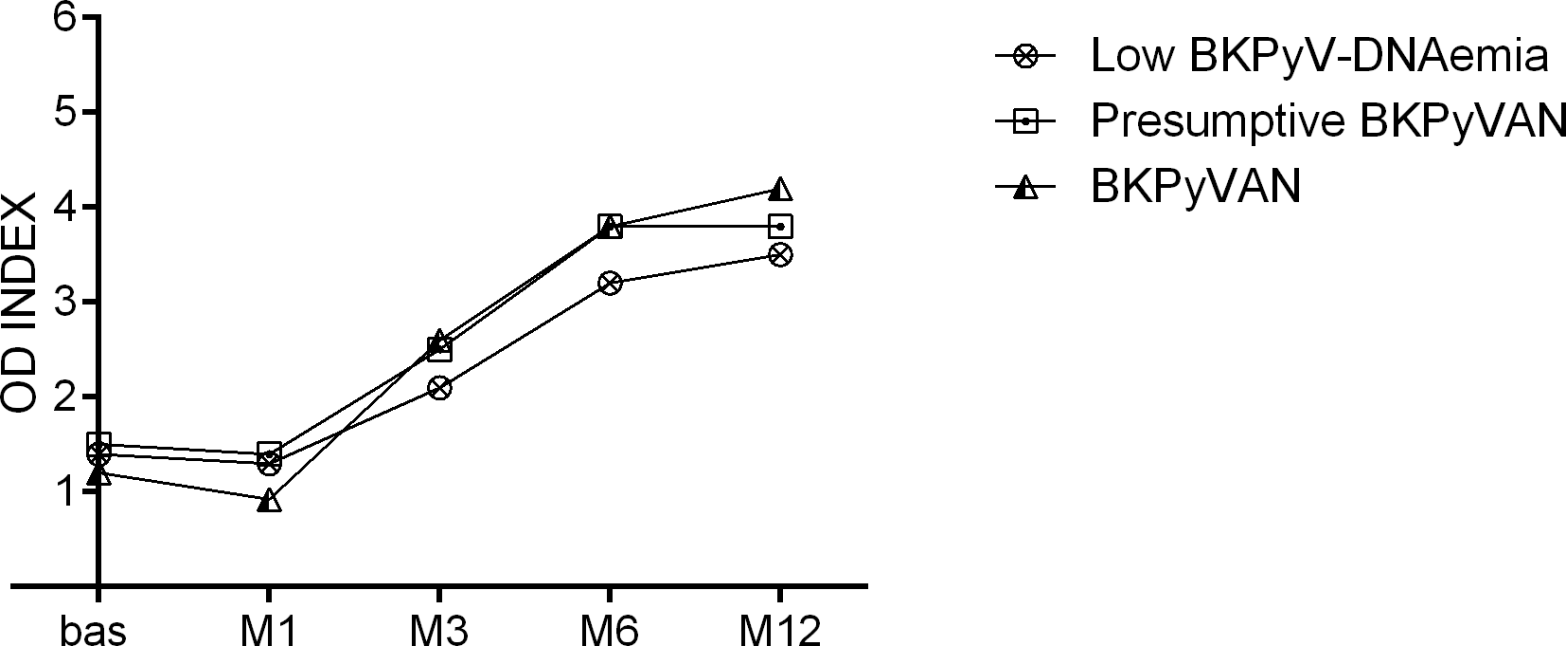
Seroreactivity during the follow-up in recipients with BKPyVAN, presumptive BKPyVAN, and low BKPyV-DNAemia. Legend: Presumptive BKPyVAN was defined as >10^4^ copies/ml, viral load <10^4^ copies/ml viremia as low BKPyV-DNAemia. BKVAN was a biopsy-proven disease.

BKPyVAN was diagnosed in 11 participants (5.2%, 11/210). Most recipients (81.8%, 9/11) with biopsy-proven BKPyVAN had peak BKPyV loads of >10^4^ copies/ml. Serological characteristics before and after transplantation were similar to those of BKPyV-DNAemic recipients. The pretransplant antibody levels were higher in the kidney donors of BKPyVAN recipients when compared to BKPyV-negative recipients (p=0.058, **Figure 5**). The seroreactivity increase in recipients during the 1 year of follow-up was highest in the BKPyVAN group, although the dynamics was similar to those of the BKPyV-DNAemia (**Figure 6**). The pretransplant seroprevalence among patients with BKPyVAN was 45.5% with the mean OD of 1.2 ± 0.81, then increased dramatically to 4.2 ± 0.57 at M12 (p=<0.0001). At the end of follow-up, all patients with BKPyVAN were seropositive. The patients with BKPyVAN had a higher mean antibody titer when compared to low BKPyV-DNAemia and presumptive BKPyVAN at the 1-year follow-up (p=0.046). None of the patients with biopsy-proven BKPyVAN lost their grafts during the follow-up. Most of the recipients who developed nephropathy had their immunosuppression therapy switched from tacrolimus to cyclosporine A or mycophenolate mofetil (81.8%, 9/11); one recipient was treated with cidofovir (9.1%, 1/11), and one patient remained with original immunosuppression (9.1%, 1/11).

No significant differences were observed between BKPyV-DNAemic, BKPyV-negative, and BKPyVAN patients regarding donor or recipient baseline characteristics, such as the underlying condition and immunosuppressive regimen. There was no statistically significant difference in the donor and recipient age, sex, HLA mismatches, and the length of dialysis treatment between the groups. Based on multivariable analyses, the presence of antibodies in donors was the strong predictor of posttransplant BKPyV-DNAemia (p=0.009). However, when considering the biopsy-proven BKPyVAN recipients separately, the predictive value was not found as that for all BKPyV viremic recipients.

## Discussion

In this retrospective study, we aimed to evaluate the role of pretransplant BKPyV-specific antibodies as a predictive marker for posttransplant BKPyV replication. The pretransplant seroprevalence and antibody levels were comparable among patients after KT who stayed BKPyV negative or developed BKPyV-DNAemia and had no effect on posttransplant BKPyV reactivation. The pretransplant seropositivity did not prevent recipients from BKPyV infection as BKPyV DNA was later detected in both pretransplant seropositive and seronegative patients. However, donor BKPyV IgG seroreactivity was strongly associated with BKPyV-DNAemia and biopsy-proven BKPyVAN after KT. Multivariate analysis confirmed the presence of anti-BKPyV-specific antibodies to be an independent predictor for the development of BKPyV-DNAemia. This suggests that donor anti-BKPyV-specific antibodies could be a good predictive marker for posttransplant BKPyV replication with relevance to risk-stratified care management of kidney transplant recipients.

BKPyV reactivation that leads to BKPyVAN is one of the main issues in the management of kidney transplant patients. As part of disease prevention, kidney transplant recipients are regularly screened for the BKPyV viral load in plasma. A plasma viral load exceeding 10^4^ copies/ml indicates the need to reduce or discontinue immunosuppression. However, such intervention poses the risk of graft rejection. A negative aspect of the routine plasma screening is that only a subset of recipients with BKPyV-DNAemia develop BKPyVAN, and some BKPyVAN cases may not reach the critical value for intervention (1). Apart from the BKPyV DNA quantification, no other screening is recommended to help identify patients at risk of disease development. Because BKPyV infection elicits robust and relatively stable antibody responses against viral capsid protein VP1, the serology assays with high sensitivity can be used to detect anti-VP1 antibodies.

In children, the absence of BKPyV-specific antibodies before transplantation is one of the risk factors for BKPyV-DNAemia and BKPyVAN (11, 12). The significance of the pretransplant seroprevalence and antibody level in adults is less clear, probably due to a higher seroprevalence of BKPyV antibodies. Some previous studies suggested that higher pretransplant antibody titers in recipients limited viral replication (13–15). Lower pretransplant anti-BKPyV antibody levels were found in recipients who developed posttransplant BKPyV-DNAemia, as opposed to recipients with BKPyV DNA in urine (14). It was shown that specific antibodies against BKPyV in serum can effectively neutralize and suppress BKPyV infection (16, 17). These results led to the use of intravenous immunoglobulin infusion in kidney transplant recipients to increase virus neutralization antibody titers (18). However, other studies including ours did not find an inverse correlation between BKV-specific IgG titers in the pretransplant recipient and posttransplant BKPyV-DNAemia (5, 6).

Unlike pretransplant antibody levels in recipients, donor antibody levels are a risk factor for BKPyV reactivation. In our study, higher antibody levels were found in kidney allograft donors for recipients who later developed BKPyV-DNAemia or BKPyVAN. We speculated that this might be associated with recent active infection in the donors. However, no donors were plasma BKPyV DNA positive at the time of organ donation. Several studies showed that high levels of donor antibodies determined alone or in combination with lower or undetectable levels of recipients’ VP1 IgG antibodies increase the risk of BKPyV-DNAemia and BKPyVAN (5, 13, 19). Our results confirmed that the serology status D+R- was associated with the highest incidence of BKPyV infection during the first year after KT. The previous study of Wunderlink showed that donor serum BKPyV IgG reactivity potentially represents an early and practical predictive biomarker of BKPyV infection reactivity in kidney transplant recipients (20). On the other hand, in agreement with other studies (5, 21), two of 30 recipients with negative serostatus (D-/R-) in our study developed BKPyV-DNAemia, suggesting that BKPyV reactivation may also occur after KT from a seronegative donor.

It is well known that posttransplant seroprevalence reflects BKPyV infection (19). Our analysis showed a significant increase in seroreactivity in kidney transplant recipients with BKPyV reactivation in plasma. The episode of BKPyV-DNAemia is of utmost importance for the immune response and leads to massive humoral seroresponse. However, we were not able to confirm the association between posttransplant BKPyV antibody levels and the development of biopsy-proven BKPyVAN. The explanation might lie in the fact that the pathology of BKPyVAN involves varying degrees of viral inclusions and different localization (interstitial inflammation/tubulitis) (22), which, in turn, may lead to differences in antibody titers and may not be recognizable in such small cases. Without any episode of BKPyV-DNAemia, the level of BKPyV Ig antibodies remained stable throughout the 1-year follow-up period. In accordance with our results, a significant increase in anti-BKPyV-specific antibodies in renal transplant patients with BKPyV-DNAemia was observed by others (13–15, 23, 24). Hariharan et al. found significantly higher levels of BKPyV-specific antibodies in patients after BKPyVAN recovery and BKPyV clearance than at the time of BKPyV detection and nephropathy diagnosis and believed that the increase in BKPyV-specific antibodies was associated with viral clearance (23). Nevertheless, our results suggest that the increased antibody levels were related to BKPyV replication itself, reflecting the viral load rather than viral clearance. Other authors reported a load-dependent relationship between BKPyV seroreactivity and BKPyV-DNAemia, assuming that BKPyV seroreactivity in immunocompetent individuals is consistent with the level of BKPyV-DNAemia experienced during infection (19).

In this study, we did not demonstrate a strong association between donor BKPyV seroreactivity and subsequent biopsy-proven BKPyVAN. The prevalence of BKPyVAN in our study was 5.2%, but none of the recipients with BKPyVAN lost the allograft. This is in contrast to the study by Wunderlink et al. (6), which found a significant correlation between the donor BKPyV-specific IgG levels and BKPyVAN even for a small number of recipients. In the previous study, the Luminex immunoassay with the BKPyV GST-VP1 antigen presented on a bead was used to detect BKPyV antibodies and compared with the ELISA with the BKPyV VP1 VLP antigen, and both methods showed good agreement in antibody detection. However, the two methods are likely to differ in the determination of the antibody level. Unlike the Luminex immunoassay, ELISA tests are generally less demanding and are routinely used. The sensitivity and specificity of ELISA tests are not always equivalent, differing in several parameters, such as the cutoff values, sample dilution, or antigens used. Since there is no standardization of antibody detection, Dakroub et al. used a commercial BKPyV VP1 antibody for interplate normalization and suggested possible applications for the commercial development of the ELISA tests (21).

A possible limitation of the study was the selection of recipients to the study based on their survival in the first posttransplant year. Patients were selected according to the availability of pretransplant plasma samples and 1 year after transplantation. Nevertheless, looking at the clinical and laboratory parameters of the excluded patients, there is no indication that the completion of the sample set would have changed the results. The low incidence of biopsy-proven BKPyVAN limited the multivariate analysis. However, the incidence rates of BKPyV-DNAemia and BKPyVAN in our study population were comparable to the data of a previous study performed in the same transplant center (25). Another limitation was the lower number of donor samples resulting from the organ allocation system. Despite the smaller number of donors, statistically significant results were obtained. Our study showed that the pretransplant serology screening of kidney allograft donors can identify recipients at risk of BKPyV reactivation/infection. The serostatus determination in kidney allograft donors is simple and reliable tool to categorize patients into high-risk and low-risk recipients. The pretransplant screening for anti-BKPyV-specific antibodies will allow the identification of individuals who need to be tested more frequently for the presence of viral DNA in the blood in order to benefit from early detection of the risk for developing BKPyVAN. A similar strategy has been already used for EBV and HCMV infections.

The presence of BKPyV-specific antibodies in donors is a very good marker for high risk of BKPyV-DNAemia, which may allow for a better stratification of kidney transplant recipients. Knowing the donor’s serostatus could be helpful in scheduling recipients for posttransplant BKPyV screening. Kidney transplant recipients from seropositive donors should be routinely screened for BKPyV DNA in plasma much more frequently than kidney transplant recipients from seronegative donors, as recommended in Hirsch and Randhawa (1).

## Data availability statement

The original contributions presented in the study are included in the article. Further inquiries can be directed to the corresponding authors.

## Ethics statement

The studies involving human participants were reviewed and approved by the ethics committee of the Institute for Clinical and Experimental Medicine in Prague (number G-16-06-27). The patients/participants provided their written informed consent to participate in this study.

## Author contributions

MS participated in the study design and data analysis and performed the BKPyV DNA detection and wrote the first version of manuscript; VL and EH performed the ELISA tests; MK collected clinical specimens and the subjects’ information; VŠ participated in the data analysis and writing the manuscript; OV participated in the study design; MW participated in the study design, collecting clinical specimens, and writing the manuscript. All authors contributed to the article and approved the submitted version.

## Funding

The study was supported by Ministry of by the Ministry of Health of the Czech Republic, grant number 17-29992A, all rights reserved.

## Acknowledgments

The authors thank Pavla Pecherková for statistical analyses and Kristýna Klečáková and Nela Václavíková for expert technical assistance.

## Conflict of interest

The authors declare that the research was conducted in the absence of any commercial or financial relationships that could be construed as a potential conflict of interest.

## Publisher’s note

All claims expressed in this article are solely those of the authors and do not necessarily represent those of their affiliated organizations, or those of the publisher, the editors and the reviewers. Any product that may be evaluated in this article, or claim that may be made by its manufacturer, is not guaranteed or endorsed by the publisher.
